# A new adult AML case with an extremely complex karyotype, remission and relapse combined with high hyperdiploidy of a normal chromosome set in secondary AML

**DOI:** 10.1186/s12878-018-0114-3

**Published:** 2018-08-31

**Authors:** Abdulsamad Wafa, Suher ALmedania, Abdulmunim Aljapawe, Thomas Liehr, Soulaiman E. Soulaiman, Raja Mouna, Moneeb A. K. Othman, Walid ALachkar

**Affiliations:** 1 Department of Molecular Biology and Biotechnology, Human Genetics Division, Atomic Energy Commission, Damascus, Syria; 2 Department of Molecular Biology and Biotechnology, Mammalians Biology Division, Atomic Energy Commission, Damascus, Syria; 3 Institute of Human Genetics, Jena University Hospital, Jena, Germany; 4 Department of Haematology-transplantation, Tishreen Hospital, Damascus, Syria

**Keywords:** Acute myeloid leukemia, Complex karyotype, High hyperdiploidy, Pentasomy 4, Molecular cytogenetics, Array comparative genomic hybridization (aCGH), Prognostic factors

## Abstract

**Background:**

Chromosomal abnormalities are diagnostic and prognostic key factors in acute myeloid leukemia (AML) patients, as they play a central role for risk stratification algorithms. High hyperdiploidy (HH), a rare cytogenetic abnormality seen commonly in elder male AML patients, is normally categorized under AML with complex karyotype (CK). Accordingly, patients with HH generally are associated with low remission rates and a short overall survival.

**Case presentation:**

Here we report a case of 21-year-old female, diagnosed with a de novo AML-M1 according to WHO classification and a CK at diagnosis. Cytogenetic, molecular cytogenetic approaches (standard fluorescence in situ hybridization (FISH), array-proven multicolor banding (aMCB)) and high resolution array comparative genomic hybridization (aCGH) analyses revealed a unique complex but still near diploid karyotype involving eleven chromosomes was identified. It included pentasomy 4, three yet unreported chromosomal aberrations t(1;2)(p35;p22), t(1;3)(p36.2;p26.2), and t(10;12)(p15.2;q24.11), and a combination of two cytogenetic events, yet unreported to appear in together, i.e. a reciprocal translocation t(1;3)(p36.2;p26.2) leading to *EVI1*/*PRDM16* gene fusion, and monoallelic loss of tumor suppressor gene *TP53*. After successful chemotherapeutic treatment the patient experienced a relapse to AML-M1, and she developed secondary AML-M6 with tetraploidy and HH. Unfortunately, the young woman died 8.5 months after initial diagnosis.

**Conclusions:**

To the best of our knowledge, a comparable adult AML associated with such a CK, coexistence of 3q rearrangements with loss of *TP53* at diagnosis, and HH in secondary AML were not previously reported. Thus, the combination of the here seen chromosomal aberrations in adult primary AML seems to indicate for an adverse prognosis.

## Background

Acute myeloid leukemia (AML) may be observed in children and/or adult patients. It is well established, that acquired chromosomal rearrangements play a central role in risk stratification of the disease [1–4]. Accordingly, the European Leukemia Net (ELN) recommendations [5] classified specific, repeatedly observable chromosomal abnormalities according to prognoses as

- favorable – e.g. t(8;21)(q22;q22), t(15;17)(q21;q21), inv.(16)(p13q22)}
- intermediate – e.g. t(9;11)(p22;q23), or
- adverse – e.g. -5 or del(5q), − 7 or del(7q), abnormalities of 3q, abnormalities of 17p, translocations t(9;22)(q34;q11), translocation t (v;11q23.3), complex karyotype (CK) and near haploid karyotype.

Approximately 10 to 15% of AML patients had a CK [1, 2, 4, 6, 7], which have been associated with a poor prognosis, but were defined differently as the presence of ≥3 and/or ≥ 5 chromosome aberrations [1, 2, 4, 6, 7]. CKs, at the cytogenetic level are very heterogeneous and many studies have suggested new definitions based on affected regions or types of aberrations [2, 8].

High hyperdiploidy (HH) (i.e. ≥49 chromosomes with or without additional structural rearrangements) is a very rare event observed in small subset of adult AML (< 2%) only [9, 10]; it is primarily seen in de novo AML and older male patients with low remission rate and short survival [9]. Interestingly, Chilton et al. [11] indicated that not all HH-AML patients should be automatically classified as having adverse prognosis. Only those patients with the presence of other specific adverse cytogenetic features (for example, − 5 or 5q-, − 7 or 7q-, abnormalities of 3q, translocation t(9;22) and certain *MLL* translocations) can confidently be assigned to the adverse risk group, whereas those with numerical changes only, should be classified into the intermediate risk group [11].

We present here for the first time a de novo adult AML case with a yet unreported complex karyotype involving eleven chromosomes at diagnosis and a subsequent tetraploidy and HH without all the previously observed changes in a secondary AML.

## Case presentation

**Fig. 1:**
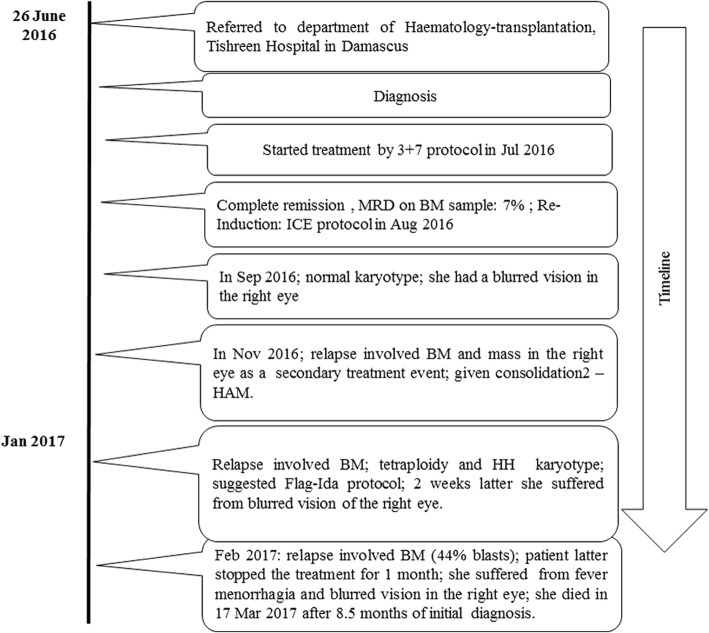
Summarizing scheme of disease progress

**Fig. 2:**
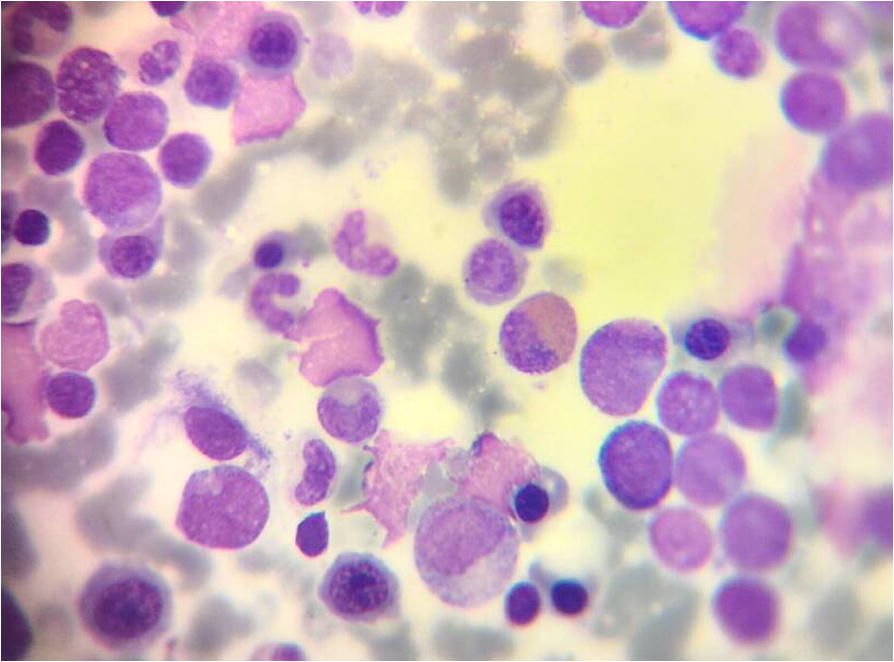
Bone marrow smears of an acute myeloid leukemia without maturation case showing numerous blasts with round nuclei, fine nuclear chromatin, and dark blue cytoplasm (Leishman stain, oil immersion × 100)

A 21-year-old female patient without any known adverse medical background presented with a 1 month history of headache, nausea, fatigue and blurred vision. Physical examination and computer tomographic (CT) scan showed pericardial inflammation and splenomegaly (2 cm). Ophthalmoscopy of the right eye revealed papillary edema, retinal hemorrhages (Roth’s spots) and arteriovenous nickings (for further details see Fig. 1 and Table 1). Initial laboratory evaluation of peripheral blood (PB) revealed a white blood cells (WBC) of 113.2 × 10^9^/l (72% were blasts), red blood cells (RBC) count was 2.53 × 10^6^/mm^3^, with a hemoglobin level of 9 g/dl and a platelet count (Plt) of 61 × 10^9^/l. Prothrombine time was 15.1 s (normal value 10.0–13.0 s) while partial thromboplastin time (PTT) was 25.8 s (normal value 29 ± 3.5 s). Creatinine value showed 38.7 μmol/l (normal 45–120) and uric acid value 498.2 μmol/l (normal 150–450). Bone marrow (BM) aspiration revealed 70% of blasts (Fig. 2).

**Table 1:** Clinical history of the patient together with diagnostic results and treatment

Date	Symptoms	Analyses on BM sample	Treatment and Outcomes
26 June 2016	- Headache, nausea, fatigue and blurred vision for 1 month ago. - Abdomen CT scan showed pericardial inflammation and splenomegaly (2 cm). - Ophthalmoscopy of the right eye revealed papillary edema, retinal hemorrhages (Roth’s spots) and arteriovenous nickings. -CT scan for brain was normal. -Peripheral blood (PB) showed: Leukocytosis (113.2 × 10^9^/l), anemia (9 g/dL), and thrombocytopenia (61 × 10^9^/L). -Bone marrow (BM) smear showed almost 70% of blats.	- Prior to chemotherapy treatment GTG-banding cytogenetics revealed a karyotype 48–50,X,-X, der(1)t(1;2)(p35;p22),der(1)t(1;3)(p36.21;p26.2),der(2)(:1p36.21- > 1p35::2p22- > 2qter),+ 4,+ 4,+ 4,+ 6,der(8)t(8;11)(q24.3;q13.4),der(10)t(10;12)(p15.3;q24.11),del(10)(q21q21),dic(12;17)(p11.2;p11.2), del(15)(q14q14), del(15)(q21.1q21.1), del(15)(q22.32q24)del(17) (q12q12) [14] - Molecular cytogenetic studies showed: confirmed the complex aberrations and a monoallelic loss of tumor suppressor gene *TP53.* - Flow cytometric (FCM) analysis of BM specimen prior to chemotherapy treatment characterized this case as AML-M1 according to WHO classifications. The abnormal cell population (60% in BM) was positive for CD45dim, CD34, HLADr, CD33, CD117, CD13 and CD11c. This cell population was negative for cyCD3, cyCD79a, CD14, CD64, CD32, CD7, CD19, CD10, and CD5. - The aCGH analysis revealed different genomic imbalances: deletion on 17q21.3; duplication of 3q26.1q29; and trisomy # 6).	26 June −02 July 2016 - (3 + 7) protocol: (Daunorubicin 60 mg/m^2^ for 3 days and Cytarabine 200 mg/m^2^ for 7 days)
10 Jul 2016	Peripheral blood (PB) showed cytopenia (WBC 0.4 × 10^9^/l), anemia (Hgb 9.5 g/dl); thrombocytopenia (Plt 12 × 10^9^/l). Serum creatinine value was 19.8 umol/l (normal 45–120) and serum total bilirubin value was 22.2 (normal value 2–21 umol/l), serum Ca^+ 2^ value 2 (2.15–2.55 mmol/l), serum Na^+^ value 132.3 (135–148 mmol/l). -BM smear showed almost 7% of blats.	–	–
26 Jul 2016	-Complete remission (CR) PB showed: WBC (6.1 × 10^9^/l), Hgb (11.7 g/dl); Plt (303 × 10^9^/l). -BM smear showed almost 5% of blats	46,XX [19]/HeH [2]	11 Aug-17 Aug 2017 Re-Induction: ICE cytrabin 200 mg/d Day1 ➔Day7 etobside 100 mg/d Day1➔Day5 idarubicin 20 mg/d Day1 ➔Day3
25 Sep 2016	-Blurred vision in the right eye (retinal detachment sensory serous). -CR -PB showed: WBC (7.4 × 10^9^/l), Hgb (11.6 g/dl); Plt (183 × 10^9^/l). -BM smear showed almost 4% of blats	46,XX [14]	26 Sep-28 Sep 2017 Consolidation1 - HAM Cytrabin 3G/m^2^/d Day1➔Day3 Methoxantron 20 mg/d D1,D2
15 Nov 2016	Relapse. -Secondary treatment event: mass under vascular arch with splint edema of optical nerve of the right eye which causes to sever decrease of vision in the right eye. -BM smear showed almost 20–30% of blats. -Cerebrospinal fluid (CSF) was negative. PB showed: WBC (5.6 × 10^9^/l, with 98.5 of neutrophils), Hgb (11.6 g/dl); thrombocytopenia [Plt (70 × 10^9^/l)]. -Serum creatinine value was 39 umol/l (normal 45–120) and serum Ca^+ 2^ value was 1.94 (2.15–2.55 mmol/l). -CT scan of brain was normal.	–	17 Nov-19 Nov 2017 Consolidation2 - HAM Cytrabin 3G/d Day1➔Day3 Methoxantron 20 mg/d D1,D2
30 Nov 2016	PB showed: Cytopenia [WBC (0.1 × 10^9^/l)], anemia [Hgb (8.4 g/dl)]; thrombocytopenia [Plt (20 × 10^9^/l)]. Serum creatinine value was 33 umol/l (normal 45–120), serum serum K^+^ value 2.89 (3.5–5.2 mmol/l), and serum Na^+^ value 134.6 (135–148 mmol/l).	–	The mass behind the retina of the right eye was still present
03 Jan 2017	-Disappeared the previous Mass behind retina. -Relapse. PB showed: WBC (7.5 × 10^9^/l, with 77.7 of neutrophils), Hgb (12 g/dl); Plt (178 × 10^9^/l). -BM smear showed almost 15% of blats	- Post to chemotherapy treatment GTG-banding cytogenetics revealed a karyotype 92,XXXX [4]/62,XX,+ 1,+ 4,+ 5,+ 5,+ 6, + 6, + 11, + 15, + 16, + 17, + 19, + 19, + 20, + 20,+ 21, + 22 [2]/46,XX [15]. FCM analysis of BM specimen post to chemotherapy treatment characterized this case as AML-M6 according to WHO classifications. The abnormal cell population (15%) was positive for CD45dim, CD36, HLADr, CD33, CD34, CD117, CD13, CD235a and MPO. Those blasts were negative for: CD10, CD19, CD20, CD22, CD5, CD7, CD2, CD3, CD16, CD56, CD1a, CD14, CD64, CD32, TdT, cyCD3 and cyCD79a.	05 Jan 2017 PB showed: WBC (3.5 × 10^9^/l, with 84.4 of neutrophils), anemia [Hgb (9.3 g/dl)]; thrombocytopenia [Plt (33 × 10^9^/l)]. 10 Jan 2017 The MD’s suggested Flag-Ida protocol. No Flag-Ida treatment available because the political situation in his country. She was given Cytrabin 100 mg per day
25 Jan 2017	-Blurred vision in the right eye (central retinal detachment serous). -PB showed: WBC 60 × 10^9^/l (70% of them were blats), Hgb 13.3 g/dl; thrombocytopenia Plt 13 × 10^9^/l. -Brain MRI was normal.	–	She was treated with: Cytrabin 1 g/d Day1➔day3 Etoposide 100 mg/d day1 ➔day3 Methoxantron 20 mg/d Day1 ➔ Day2
13 Feb 2017	PB showed: Cytopenia [WBC (0.5 × 10^9^/l)], anemia [Hgb (9.7 g/dl)]; thrombocytopenia [Plt (13 × 10^9^/l)]. Serum creatinine value was 34 umol/l (normal 45–120),serum serum K^+^ value 2.92 (3.5–5.2 mmol/l), serum Na^+^ value 134.9 (135–148 mmol/l), and serum total bilirubin value was 24.09 (normal value 2–21 umol/l).	–	16 Feb 2017 PB showed: Cytopenia [WBC (1.5 × 10^9^/l, with 44% of them were basts)], anemia [Hgb (9.6 g/dl)]; thrombocytopenia [Plt (17 × 10^9^/l)]. Serum creatinine value was 34 umol/l (normal 45–120),serum serum K^+^ value 2.57 (3.5–5.2 mmol/l), serum and Na^+^ value 134.1 (135–148 mmol/l).
17 Mar 2017	-Her MD’s stooped her treatment depended on her request from 1 month. -Relapse. -BM smear showed almost 44% of blats. -PB showed: WBC (7.5 × 10^9^/l, with 77.7 of neutrophils), Hgb (12 g/dl); Plt (178 × 10^9^/l).		- She suffered from fever more than 40 C° for more than 3 days, menorrhagia and blurred vision in the right eye. -Approximately 8.5 months after initial diagnosis she died due to unknown causes. -No autopsy was performed because she died in her house.

At this point the first cytogenetic and immunophenotypic data were determined. Flow cytometric (FCM) analysis classified this case as AML-M1. The patient was given standard treatment for AML including (3 + 7) induction chemotherapy (Daunorubicin 60 mg/m^2^ for 3 days and Cytarabine 200 mg/m^2^ for 7 days). On day + 28 of treatment with (3 + 7) protocol, the patient had not responded as expected to the treatment, i.e. her PB revealed pancytopenia/cytopenia (WBC 0.4 × 10^9^/l), anemia (hemoglobin level = Hgb: 9.5 g/dl); thrombocytopenia (Plt 12 × 10^9^/l) and less than 7% blasts in BM aspiration. The patient was given re-induction chemotherapy (ICE protocol: Cytrabin 200 mg/day: day 1 ➔ day 7, Etobside 100 mg/day: day 1 ➔ day 5, and Idarubicin 20 mg/day: day 1 ➔ day 3) and she achieved complete remission on day 30 of ICE protocol treatment (WBC 7.4 × 10^9^/l; Hgb 11.6 g/dl; Plt 183 × 10^9^/l), with less than 4% blasts in BM aspiration. Still the patient suffered from blurred vision in the right eye (retinal detachment sensory serous) during ICE protocol treatment but her karyotype was normal. The patient was given consolidation I chemotherapy (High dose Ara-C = HIDAC: Cytarabine 3 g/m^2^/day; day 1 ➔ day 3; and Methoxantron 20 mg/day; day 1 ➔day 2). Afterwards the patient did not return to the hospital to continue the treatment for 6 weeks. Then she was referred to the hospital again for blurred vision in the right eye and a mass under the vascular arch with splint edema of optical nerve of the right eye was diagnosed, being the cause of her severe decrease in vision. While cerebrospinal fluid (CSF) test was negative, BM aspiration revealed 20–30% of blasts. In PB WBC was 5.6 × 10^9^/l (98.5% of neutrophils), Hgb was 11.6 g/dl, Plt of 70 × 10^9^/l indicated for thrombocytopenia while CT scan of brain was normal. Now she treated with consolidation II chemotherapy (HIDAC), 2 weeks later her PB had WBC 0.1 × 10^9^/l, Hgb 8.4 g/dl and Plt still 20 × 10^9^/l; the mass behind the retina of the right eye was still present.

About 2 months later the patient relapsed and the following values were found: in PB WBC was 7.5 × 10^9^/l with 77.7% of neutrophils, Hgb 12 was g/dl and Plt was 178 × 10^9^/l; BM aspiration revealed 15% of blasts. The MD’s suggested to apply now the Flag-Ida protocol; however, due to the political situation in her home country only available treatment at this point was treatment with Cytrabin 100 mg/day. Again 2 weeks later the patient suffered from blurred vision of the right eye due to serious central retinal detachment; her PB revealed a WBC of 60 × 10^9^/l (70% of them were blasts), Hgb of 13.3 g/dl; thrombocytopenia with Plt of 13 × 10^9^/l was present with a normal brain MRI. Now the patient treated with Cytrabin 1 g/day: day 1 ➔ day 3, Etoposide 100 mg/day: day 1 ➔ day 3, and Methoxantron 20 mg/day: day 1 ➔day 2).

Ten days later, the patient relapsed; her PB shows cytopenia [WBC 1.5 × 10^9^/l with 44% blasts)], anemia (Hgb 9.6 g/dl) and thrombocytopenia (Plt 17 × 10^9^/l). Now the patient stopped the treatment on her own request for 1 month. Afterwards she suffered from fever (more than 40 °C for more than 3 days), menorrhagia and blurred vision in the right eye. Approximately 8.5 months after initial diagnosis she died in her house and no autopsy was performed. Her husband agreed with scientific evaluation of her case and the study was approved by the ethical committee of the Atomic Energy Commission, Damascus, Syria.

Conventional cytogenetics analysis on unstimulated BM sample according to standard procedures was performed [12] prior and post chemotherapy treatments. Karyotypes according to the International System for Human Cytogenetic Nomenclature were classified [13].

**Fig. 3:**
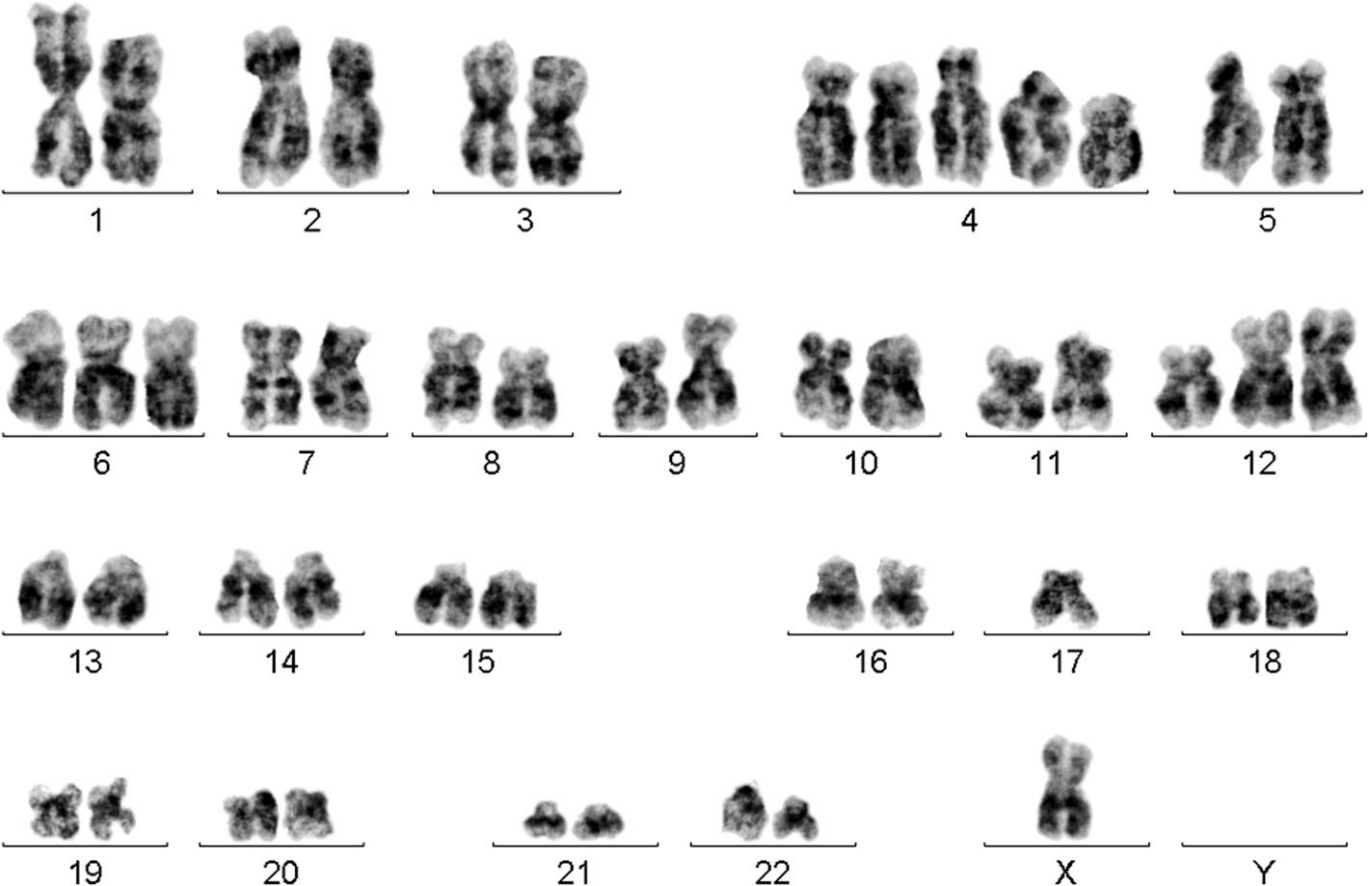
GTG-banding revealed a hyperdiploid karyotype multiple numerical and or structural rearrangements

**Fig. 4:**
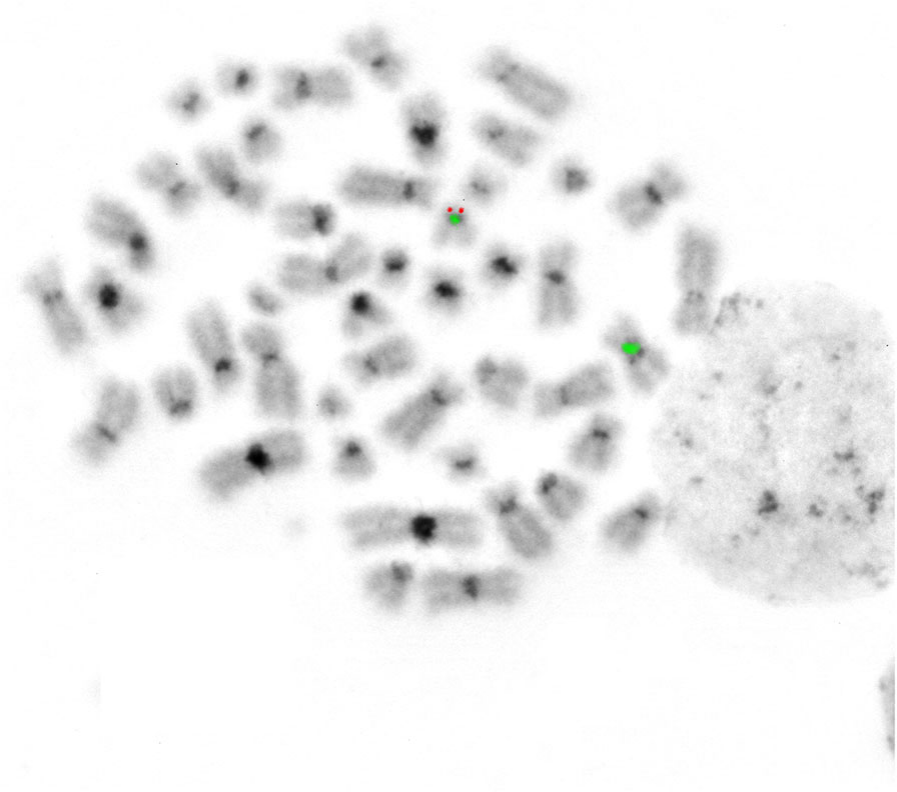
FISH result after application of probes for centromere 17 (CEP 17 green) and *TP53* gene (red) revealed a normal chromosome 17 and a derivative chromosome 17 with deletion of *TP53* gene region. Abbreviations: # = chromosome; der = derivative chromosome

**Fig. 5:**
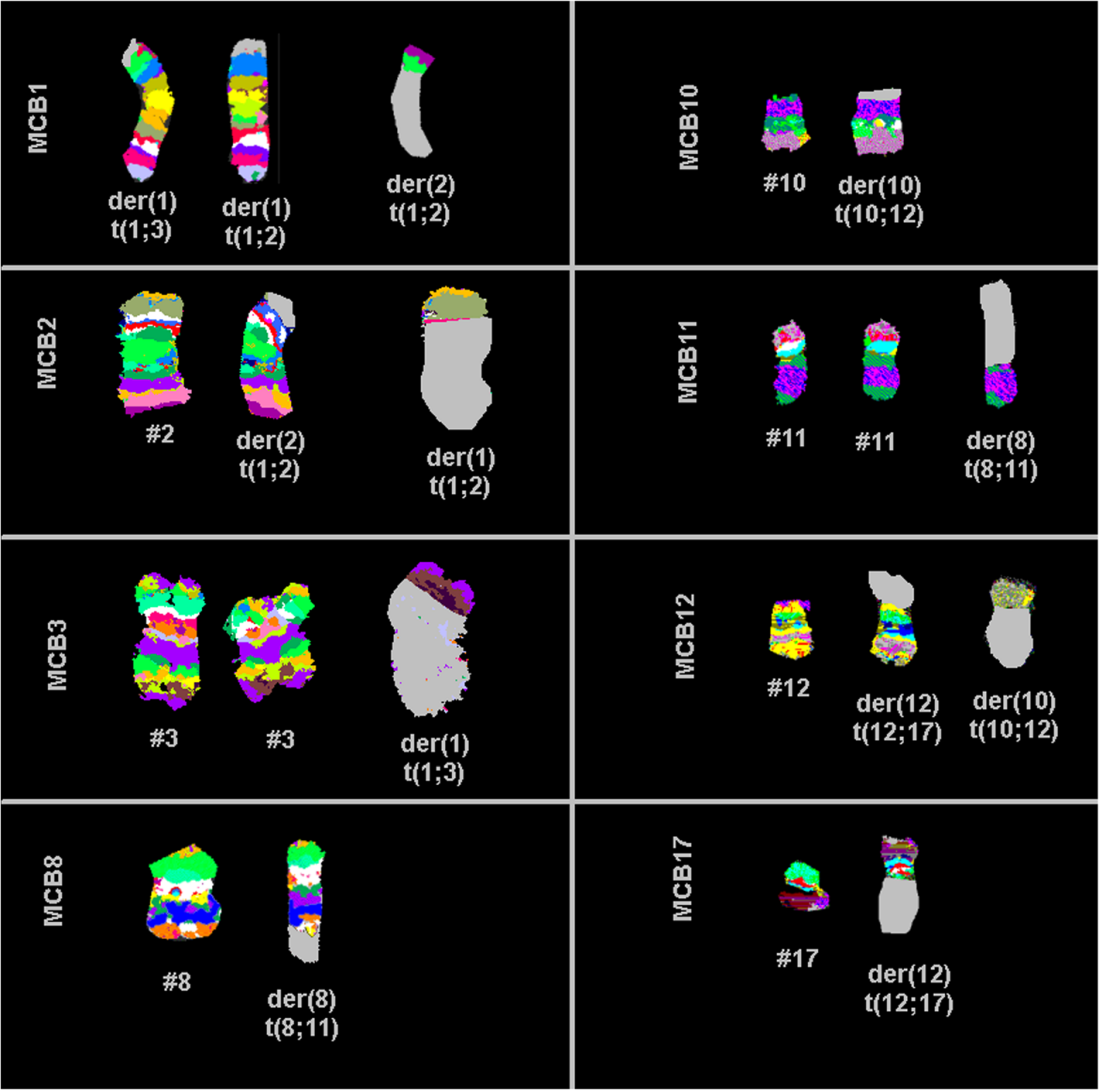
aMCB results are shown. If available, the normal chromosomes (#) are depicted on the left side and the derivative of the corresponding chromosomes on the right side of normal chromosomes. The unstained regions when using chromosome-specific aMCB-probe sets on the derivative chromosomes are shown in gray. # = chromosome; der = derivative chromosome

Prior to chemotherapy treatment: GTG-banding cytogenetics revealed the following karyotype:

- 48–50,X,- X,der(1)t(1;2)(?;?),der(1)t(1;3)(?;?),+ 4,+ 4,+ 4,+ 6,t(8;11)(?;?),t(10;12)(?;?),dic(12;17)(?;?)× 2 [14] (Fig. 3), which was further specified by molecular cytogenetic studies (Figs. 4 and 5). Fluorescence in situ hybridization (FISH) using (WCP) probes for chromosomes 1, 2, 3, 4, 5, 6, 9, 12, 17 and X (MetaSystems, Altlussheim, Germany), a specific probe for *ETV6* break apart probe and a specific probe for 17p13 (*TP53*) (Q-Biogene, USA) were applied according to manufacturer’s instructions. Array-proven multicolor banding (aMCB) probes sets for chromosomes 1, 2, 3, 8, 10, 11, 12 and 17 were used [12]. Thus, the following final karyotype prior to chemotherapeutic treatment was determined using a fluorescence microscope [12].
- 48–50,X,-X,der(1)t(1;2)(p35;p22),der(1)t(1;3)(p36.21;p26.2),der(2)(:1p36.21- > 1p35::2p22- > 2qter),+ 4,+ 4,+ 4,+ 6,der(8)t(8;11)(q24.3;q13.4),der(10)t(10;12)(p15.3;q24.11),del(10)(q21q21),dic(12;17)(p11.2;p11.2),del(15)(q14q14),del(15)(q21.1q21.1),del(15)(q22.32q24)del(17)(q12q12) [14].

**Fig. 6:**
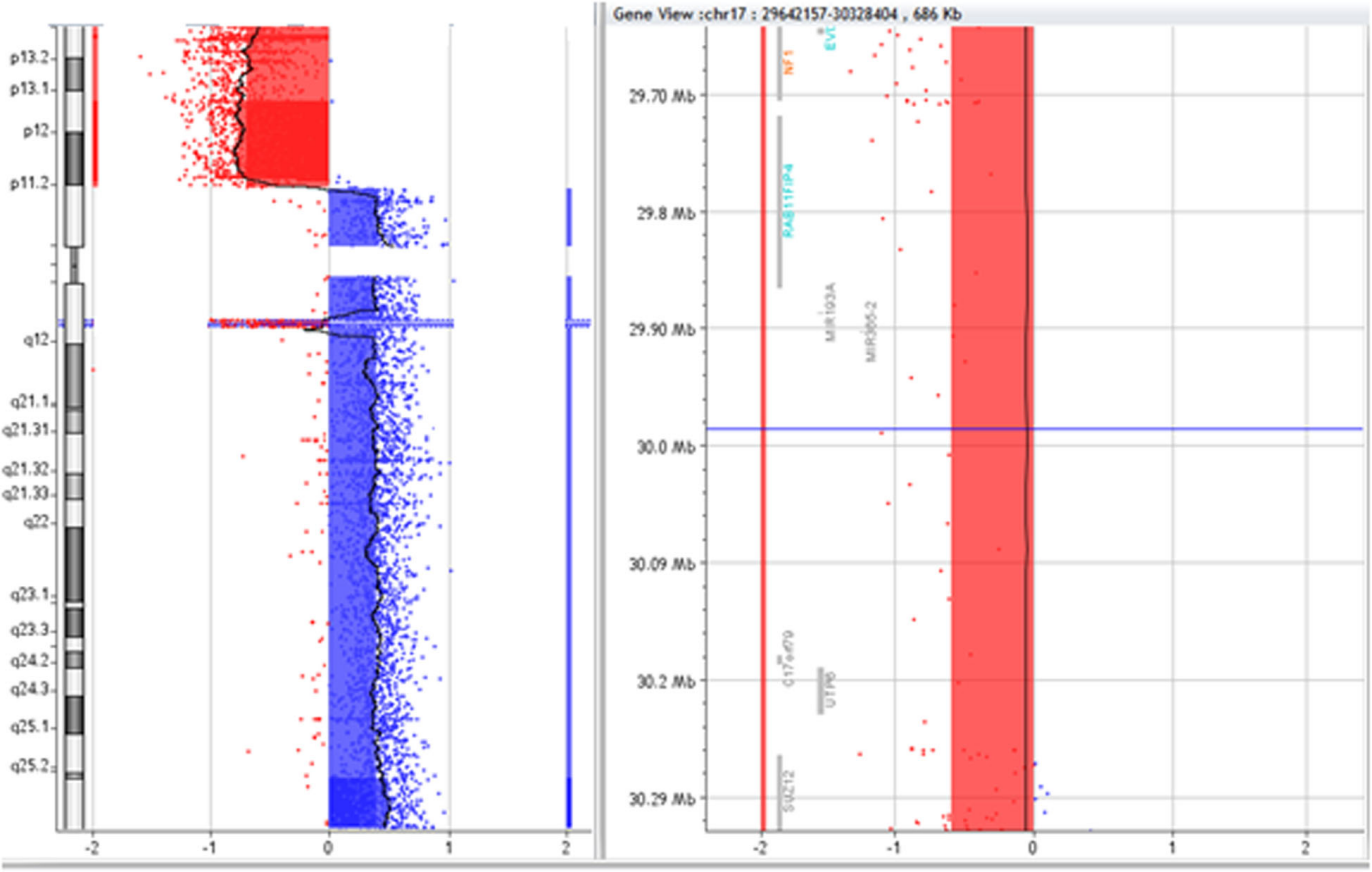
aCGH characterized losses in 17p13.3-17p11.2 and 17q11.2-17q11.2 regions and gains in 17p11.2-17p11.1, 17q11.1-17q11.2 and 17q11.2-17q12.2 regions. These observations were compatible with the FISH results and their locations according to the Genome Reference Consortium human genome (build 37) (GRCh37)/Human Genome Issue 19 (available from https://genome.ucsc.edu)

Genomic DNA was extracted from BM cells prior to chemotherapy treatment as previously reported [15]. aCGH was performed using the Agilent Sure Print G3 Human Genome Microarray 180 K as previously described [15]. The aCGH analysis revealed different genomic imbalances (Fig. 6). Thus, copy number alterations (CNAs) could be grouped according to their sizes as follows:

Focal CNAs (e.g. deletion on 14q14.3); CNAs involving variable numbers of genes (e.g. deletion on 17q21.3); CNAs involving large parts of chromosomal p or q arms (e.g. duplication of 3q26.1q29) and CNAs of whole chromosomes (e.g. trisomy # 6 -Table 2).

**Table 2:** Summary of CNAs detected by aCGH

Chromosome	Cytobands	GRCH37/hg19	Size of imbalance [Mb]
Chr. 1	del(1)(p36.33p36.22)	chr1:811,042-15,945,281	15.2
Chr. 3	dup(3)(q12.2q12.2)	chr3:100,360,692-100,444,109	0.8
	dup(3)(q26.1q29)	chr3:163,428,815-198,007,542	34.5
Chr. 4	+ 4,+ 4	+ 4	191.1
Chr. 6	+ 6	+ 6	171.1
Chr. 11	del(11)(q14.2q14.3)	chr11:88,758,551-90,262,511	1.5
	dup(11)(q24.3q25)	chr11:128,741,710-134,945,165	6.2
Chr. 12	del(12)(p13.3p11.2)	chr12:189,587-28,540,069	28.6
	dup(12)(p11.2q12.2)	chr12:29,301,936-133,783,697	104.5
Chr. 14	del(14)(q24.3q24.3)	chr14:78,947,104-78,999,179	0.52
Chr. 15	del(15)(q14q14)	chr15:35,834,701-38,130,638	2.3
	del(15)(q21.1q21.1)	chr15:45,686,828-49,092,091	3.4
	del(15)(q23q24.2)	chr15:69,669,842-75,954,617	6.3
Chr. 17	del(17)(p13.3p11.2)	chr17:6011-16,229,582	16.2
	dup(17)(p11.2p11.1)	chr17:16,387,310-22,226,321	5.8
	dup(17)(q11.1q11.2)	chr17:25,300,199-29,639,240	4.3
	del(17)(q11.2q11.2)	chr17:29,642,157-30,328,404	0.7
	dup(17)(q11.2q12.2)	chr17:30,426,721-81,044,553	50.6
Chr. 19	del(19)(q13.2q13.31)	chr19:43,242,795-43,629,732	0.4
Chr. X	-X	-X	155.0

Immunophenotyping was performed on BM specimen prior and after chemotherapy treatment using a general panel of fluorescent antibodies against antigens typical for different cell lineages and cell types [16]: CD1a, CD2, CD3, CD4, CD5, CD8, CD10, CD11b, CD11c, CD13, CD14, CD15, CD16, CD19, CD20, CD22, CD23, CD32, CD33, CD34, CD36, CD38, CD41a, CD45, CD56, CD57, CD64, CD79a, CD103, CD117, CD123, CD138, CD209, CD235a and CD243; In addition to antibodies to Kappa and Lambda light Chains, IgD, sIgM, and HLADr. All antibodies were from BD Biosciences. Flow cytometric data acquisition and analysis were conducted [17]. FCM analysis of BM specimen prior to chemotherapy treatment characterized this case as AML-M1 according to WHO classifications. The abnormal cell population (60% of tested cells) was positive for CD45^dim^, CD34, HLADr, CD33, CD117, and CD13. Blast cell population was negative for CD3, CD79a, CD14, CD64, CD32, CD7, CD19, CD10 and CD5.

**Fig. 7:**
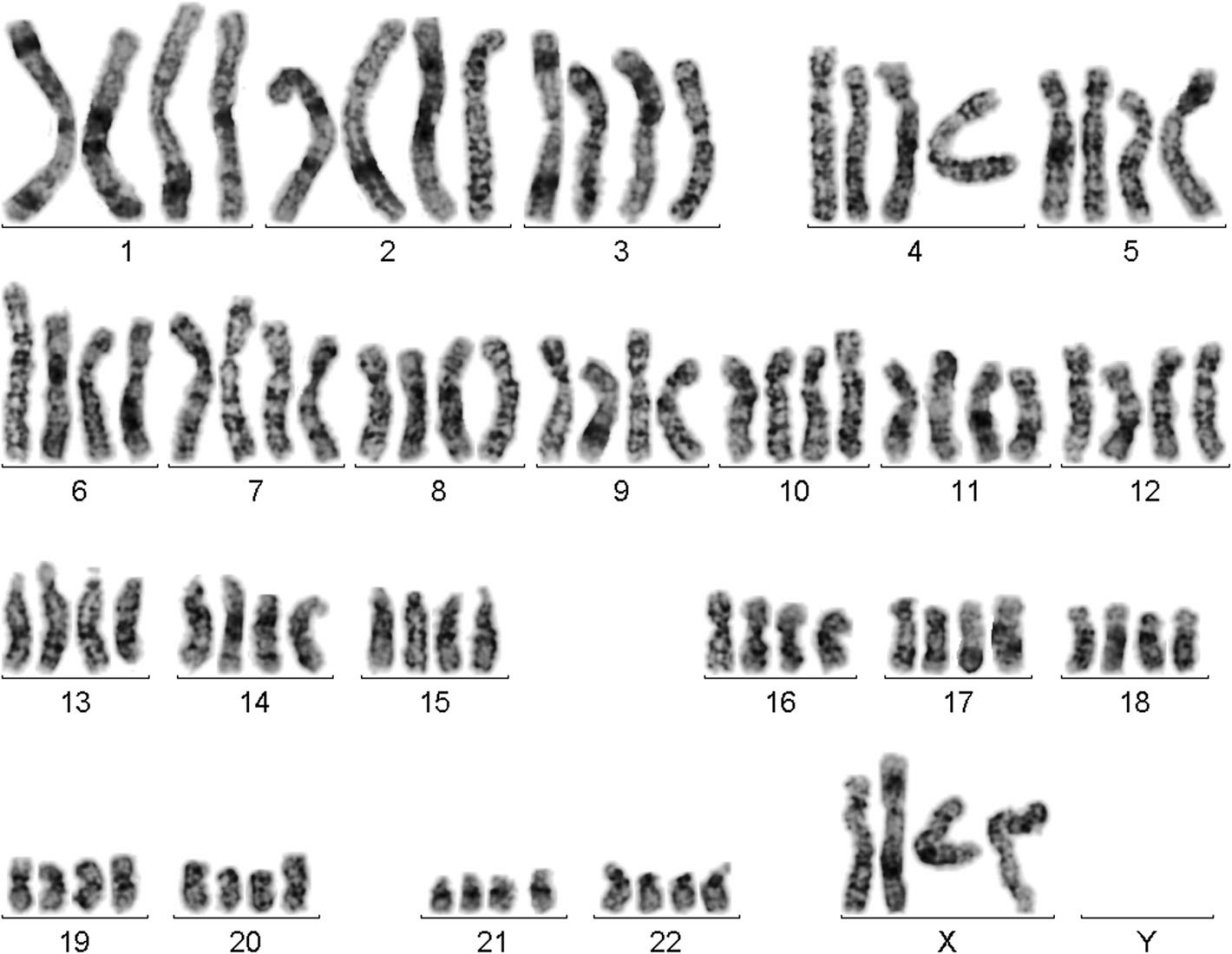
GTG-banding in secondary AML-M6 revealed a tetraploid karyotype in 20% of the analyzed cells

**Fig. 8:**
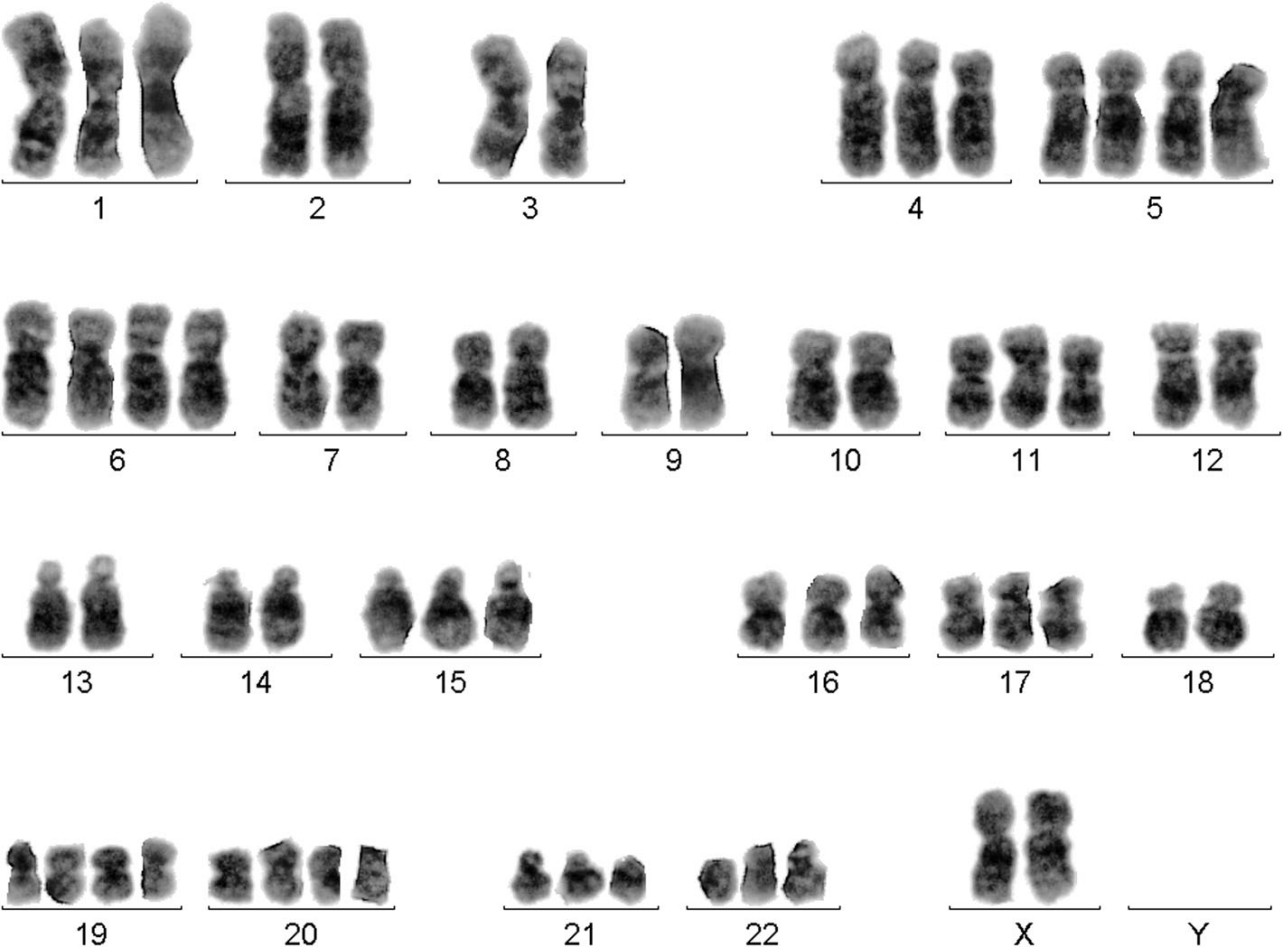
GTG-banding secondary AML-M6 revealed a hyperdiploid karyotype in 10% of the analyzed cells

After chemotherapy and relapse GTG-banding revealed a mosaic of tetraploidy and HH as 92,XXXX [4]/62,XX,+ 1,+ 4,+ 5,+ 5,+ 6,+ 6,+ 11,+ 15,+ 16,+ 17,+ 19,+ 19,+ 20,+ 20,+ 21,+ 22 [2]/46,XX [15] (Figs 7 and 8).

FCM analysis of BM specimen post to chemotherapy treatment characterized this case as AML-M6 according to WHO classifications. The abnormal cell population (15%) was positive for CD45^dim^, CD36, HLADr, CD33, CD34, CD117, CD13, CD235a and MPO. Those blasts were negative for: CD10, CD19, CD20, CD22, CD5, CD7, CD2, CD3, CD16, CD56, CD1a, CD14, CD64, CD32, TdT, cyCD3 and cyCD79a.

## Discussion and conclusions

To the best of our knowledge we report here the first case of a patient with an AML-M1 relapsing with a secondary AML-M6. In AML-M1 the patient presented a CK involving eleven chromosomes and yet unreported acquired chromosomal aberrations, while in AML-M6 a completely different, two-clonal karyotype with tetraploidy and HH was observed.

According to the literature, HH (≥49 chromosomes) and tetraploidy (4n = 92 chromosomes) has been reported to date in 15 and 99, respectively, of 18,334 AML cases listed in Mitelman database [18]. A translocation t(1;2) involving short and/or long arms of these chromosomes has been seen to date in 38 AML cases [18]. Also, deletion a part of the short arm of derivative chromosome 17, translocation t(1;3), translocation t(8;11), translocation t(10;12), deletion del(10)(q21), del(15)(q21), del(15)(q22q24) and dic(12;17) were previously reported in 3, 91, 10, 18, 1, 4, 1 and 7 AML cases, respectively [18]. Also, tetrasomy of chromosomes 4, 6, 19 and 20 were previously reported in 4, 18, 22 and 7 AML cases, respectively [18]. Interestingly, translocation t(1;2)(p35;p22), t(1;3)(p36.2;p26.2), t(10;12)(p15.2;q24.11), del(17)(q12q12), and pentasomy of chromosome 4 have never been described in AML cases. To the best of our knowledge, a combination of all these rearrangements in one AML case at diagnosis was not previous reported yet, also [18].

Gains of chromosomes, in particular tetrasomies 4, 8, 13, 14, 20 and 21, as well as pentasomies 13, 21 and 22, have been observed in AML rarely. However, there was no influence on survival observed according to the number or types of trisomies or tetrasomies [19]. Also, tetraploidy (4n, 92 chromosomes) has not previously been reported in secondary AML cases; only Harrison et al. [20] described a hypotetraploid case in a secondary AML, which had an adverse outcome.

In general, HH and tetraploidy appears infrequently in AML; it seen primarily in de novo disease in older male patients (> 60 years) with low remission rates and short overall survival (OS) [9, 10]. Unfortunately only limited data on incidence and clinical implications of HH and tetraploidy in AML is available. Still, most of comparable morphologically characterized AML cases were FAB types M2, M4, or M5 [14]. However, HH and tetraploidy was associated with poor outcome, i.e. median OS of and tetraploidy was 1.4 and for HH patients 0.6 years, which is in a similar range of CK patients with AML [14]. However, HH and tetraploidy patients with only numerical changes have a median OS of 1.0 year, while OS was 1.1 years for HH and tetraploidy patients with known non-adverse structural aberrations compared to 0.8 years for those patients with known adverse abnormalities [14]. Additionally, AML patients with ≥3 three unrelated aberrations had a worse outcome than normal karyotype patients [19]. Thus, it was repeatedly suggested in contrast to main stream [1, 5, 6], to reclassify AML patients in risk categories according to chromosomal aberrations rather than e.g. only HH [11; 18]. Stölzel et al. [19] proposed to distinguish HH patients with up to three aberrations without specific adverse-risk abnormalities, from those with more than 4 aberrations.

Concerning aberrations observed in the present case there was specifically in AML-M1 monoallelic losses for *TP53*, *ETV6*, *BRCA1* genes and or gain of copy numbers for *EVI1* (ecotropic viral integration site-1) gene. *TP53* gene mutation is observed in approximately 5–10% of all AML cases, occurring frequently in elderly subjects and cases with FAB classification M6, as well as in cases with CK; it is associated with unfavorable prognosis [21]. Aberrant expression of *EVI1* gene occurs in approximately 6–8% of AML cases and has been associated with poor treatment outcome [22, 23]. The *EVI1* gene maps to chromosomal band 3q26.2 and was first identified to be aberrantly upregulated in almost all AML cases with t(3;3)(q21;q26.2) [17] or inv.(3)(q21q26.2) [24, 25]. In our case with the t(1;3)(p36.21;p26.2), the *EVI1* locus at 3q26 is translocated to *PRDM16* (*MEL1*; *MDS1*/*EVI1*-like-1) at 1p36, being highly homologous to *EVI1* (*PRDM3*) [26]. In concordance with the conditions seen in the present case t(3;v)(q26;?) translocation was associated with younger age AML; here, the complete remission rate has been reported to be < 50% and long-term OS < 10% [25].

According to the literature the here observed, we report the first AML-M1 case relapsing to a completely independent biclonal secondary AML-M6 case. Adverse outcome of the case may be partially caused by adverse mutations in AML-M1 like *TP53* deletion and translocation t(1;3)(p36.2;p26.2) involving *EVI1* gene, but also by HH. ICE therapy might have been helpful here, however, due to interrupted treatment this cannot be finally assessed.

## Abbreviations

aCGH: Array comparative genomic hybridization
aMCB: Array-proven multicolor banding
AML: Acute myeloid leukemia
BM: Bone marrow
CK: Complex karyotype
CSF: Cerebrospinal fluid
CT: Computer tomographic
DAPI: 4′,6- diamino-2-phenylindole
ELN: European Leukemia Net
FAB: French American British
FCM: Flow cytometric
FISH: Fluorescence in situ hybridization
HGB: Hemoglobin level
HH: High hyperdiploidy
OS: Overall survival
PB: Peripheral blood
PLT: Platelet count
PTT: Partial thromboplastin time
RBC: Red blood cells
WBC: White blood cells
WCP: Whole chromosome painting
